#  Constitutive activation of MEK5 promotes a mesenchymal and migratory cell phenotype in triple negative breast cancer 

**DOI:** 10.18632/oncoscience.535

**Published:** 2021-05-18

**Authors:** Margarite D. Matossian, Van T. Hoang, Hope E. Burks, Jacqueline La, Steven Elliott, Courtney Brock, Douglas B. Rusch, Aaron Buechlein, Kenneth P. Nephew, Akshita Bhatt, Jane E. Cavanaugh, Patrick T. Flaherty, Bridgette M. Collins-Burow, Matthew E. Burow

**Affiliations:** ^1^ Department of Medicine, Division of Hematology and Oncology, Tulane University, New Orleans, LA 70118, USA; ^2^ Center for Genomics and Bioinformatics, Indiana University, Bloomington, IN 47405, USA; ^3^ Medical Sciences Program, Indiana University School of Medicine-Bloomington, Bloomington, IN 47405, USA; ^4^ Department of Pharmacology, Duquesne University School of Pharmacy, Pittsburgh, PA 15282, USA; ^5^ Department of Medicinal Chemistry, Duquesne University School of Pharmacy, Pittsburgh, PA 15282, USA; ^6^ Tulane Cancer Center, New Orleans, LA 70112, USA; ^*^ These authors contributed equally to this work and are shared first authors.

**Keywords:** triple negative breast cancer, MEK5, epithelial to mesenchymal transition, cell migration, ERK5

## Abstract

Triple negative breast cancer (TNBC) is an aggressive subtype of breast cancer with limited targeted therapeutic options. A defining feature of TNBC is the propensity to metastasize and acquire resistance to cytotoxic agents. Mitogen activated protein kinase (MAPK) and extracellular regulated kinase (ERK) signaling pathways have integral roles in cancer development and progression. While MEK5/ERK5 signaling drives mesenchymal and migratory cell phenotypes in breast cancer, the specific mechanisms underlying these actions remain under-characterized. To elucidate the mechanisms through which MEK5 regulates the mesenchymal and migratory phenotype, we generated stably transfected constitutively active MEK5 (MEK5-ca) TNBC cells. Downstream signaling pathways and candidate targets of MEK5-ca cells were based on RNA sequencing and confirmed using qPCR and Western blot analyses. MEK5 activation drove a mesenchymal cell phenotype independent of cell proliferation effects. Transwell migration assays demonstrated MEK5 activation significantly increased breast cancer cell migration. In this study, we provide supporting evidence that MEK5 functions through FRA-1 to regulate the mesenchymal and migratory phenotype in TNBC.

## INTRODUCTION

Triple negative breast cancer (TNBC) represents a particularly aggressive subtype of breast cancer. Defined by a lack of targetable receptors, treatment options for patients presenting with TNBC are limited to cytotoxic agents, such as anthracyclines and taxanes [1, 2]. Although TNBCs are initially responsive to these therapies, the risk of relapse for TNBC patients is much higher than that of women with hormone receptor positive breast cancer, with an overall worse outcome [2]. Heterogeneity within TNBC partially contributes to acquired resistance to cytotoxic agents [1, 2]. This chemotherapeutic resistance, both primary and acquired, remains a significant challenge in the clinic. Given its clinical importance, there is particular interest in determining new therapeutic targets against chemoresistance, particularly in the context of TNBC, a cancer subtype that lacks receptor-based targeted treatments [1-3]. 

Aberrant MAPK signaling has been broadly shown to mediate chemoresistance in numerous malignancies [4-7]. Activation of MAPK pathways through either mutation or direct activation promotes expression of cell survival genes and inhibits apoptosis [4, 7]. Although anti-MEK1/2 targeted therapies have been generated, the inevitable development of secondary mutations prevents the effective long-term use of these drugs in treatment regimens [7]. However, the MAP2K5, or MEK5, pathway is less characterized than other MAPK signaling pathways, and may offer an alternative target. Our lab and others have characterized MEK5/ERK5 signaling as a driver of epithelial to mesenchymal transition (EMT), resistance to apoptosis, and cell survival [8, 9]. Specifically, we have demonstrated that MEK5/ERK5 signaling mediates progression to a mesenchymal and endocrine therapy-resistant phenotype [10] and knockdown of this pathway suppresses growth and metastasis of MDA-MB-231 tumors [11]. We have also previously shown that activated ERK5 has elevated expression in breast tumors compared to adjacent normal tissue [10]. With respect to metastasis, activated ERK5 had elevated expression in brain metastases from clinically aggressive breast tumors [12]. The MEK5/ERK5 pathway regulates transcription factors that mediate the EMT phenotype including phosphorylation and increased activation of c-Fos and Fra-1 [8-10, 13, 14]. However, more extensive characterization of this relationship is needed. While Fra-1 expression activates a mesenchymal phenotype in breast cancer [15], further data is required to further support a direct relationship between MEK5/ERK5 signaling and the Fra-1-driven EMT phenotype in breast cancer. 

EMT plays an integral role in regulating various processes crucial to development, progression and recurrence of breast tumors, including metastasis, maintenance of breast cancer stem cells and acquisition of drug resistance [15-20]. In breast cancer cells, MEK5 overexpression promoted a TNFα resistance phenotype [9, 21]. Activation of the MEK5 pathway has been shown to confer a survival advantage to colon cancer cells when treated with the pyrimidine analog 5-fluorouracil (5-FU) [22]. In TNBC cells, ERK5 pharmacologic inhibition amplified anti-cancer effects of cytotoxic chemotherapies Taxotere, vinorelbine and cisplatin [23]. Conversely, ERK5 mRNA expression is associated with poor regression free survival in breast cancer patients receiving chemotherapy [24].

Characterizing signaling pathways that mediate EMT in breast cancer biology provides a promising avenue for novel therapies. Given the clinical relevance and significant regulatory roles of the MEK5/ERK5 signaling pathway in breast cancer, it is a promising therapeutic target [22, 25, 26]. Here, we present supporting evidence that constitutive activation of MEK5 drives a mesenchymal and migratory TNBC phenotype in TNBC cells.

**Figure 1:**
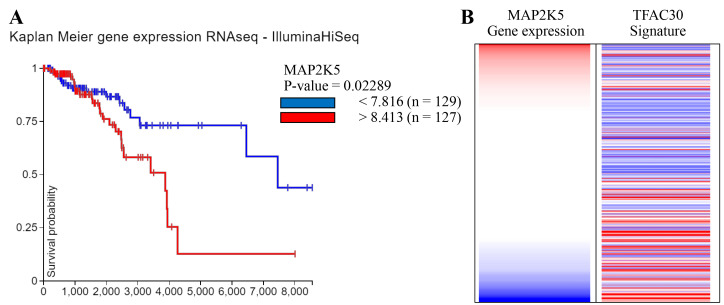
MEK5 (MAP2K5) gene expression associated with worse overall survival. **(A)** Kaplan-Meier survival plot of MEK5 (MAP2K5) gene expression extrapolated from available RNAseq data (IlluminaHiSeq) demonstrating higher MEK5 expression is associated with worse survival probability. **(B)** MEK5 (MAP2K5) gene expression is inversely correlated to the TFAC30 gene signature for complete pathologic response to cytotoxic drug therapies. Data was obtained from (A) the Kaplan-Meier plotter and (B) the TCGA databases.

## Results

### MEK5 expression is associated with worse patient survival 

 First, we performed a Kaplan Meier gene expression analysis with MEK5 (MAP2K5) to investigate effects of MEK5/ERK5 in a clinical setting. We observed that increased MEK5 expression was associated with reduced patient survival probability in breast cancer (Figure 1A). Metastasis is closely associated with therapeutic response and survival in TNBC. We examined the patterns of MEK5 expression in patient data using the Xena browser and TCGA cancer browser. The TFAC30 gene signature was generated by Hess *et al*., and lists 30 genes whose gene expression profile is predictive of complete pathologic response to chemotherapy treatment in breast cancer [27]. This signature is high in the basal subtype and ER negative patient samples. This gene signature is as follows: E2F3 + MELK + RRM2 + BTG3 - CTNND2 - GAMT - METRN - ERBB4 - ZNF552 - CA12 - KDM4B - NKAIN1 - SCUBE2 - KIAA1467 - MAPT - FLJ10916 - BECN1 - RAMP1 - GFRA1 - IGFBP4 - FGFR1OP - MDM2 - KIF3A - AMFR - MED13L - BBS4. Elevated MAP2K5 gene expression in breast cancer patients across all subtypes inversely correlated with the TFAC30 gene signature (Figure 1B). Together, these data are in line with previous studies which have investigated the MEK5 signaling axis in breast cancer outcomes using the Kaplan Meier analyses and strengthen the importance of MEK5 signaling in breast cancer progression and outcomes. 

### MEK5 drives EMT and cell migration

**Figure 2:**
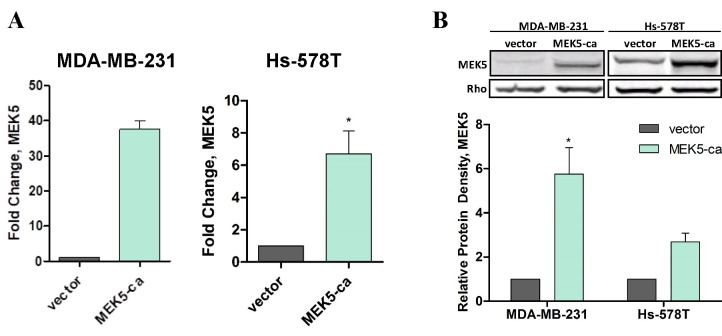
Confirmation of MEK5-ca cells. MDA-MB-231 and Hs-578T cells were transfected with vector or MEK5DD plasmid. Cells were treated with selectable marker (puromycin). Viable colonies were cloned and pooled for analysis. qPCR was performed on (A) MDA-MB-231- and Hs-578T-vector and -MEK5-ca cells for MEK5 expression. (B) Total protein was extracted from TNBC-MEK5-ca cells and western blot was performed for total MEK5 expression. Rho-GDIα served as a loading control. Bars represent normalized protein density ± SEM and vector-control cells set to 1, n ≥ 3. * p < 0.05; ** p < 0.01.

**Figure 3:**
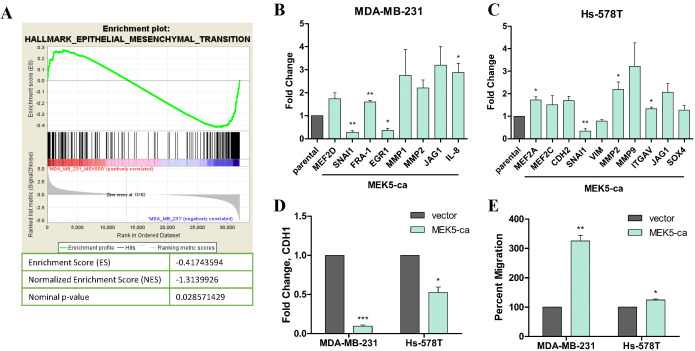
Constitutive activation of MEK5 downregulates CDH1 expression and enhances TNBC cell migration. **(A)** GSEA analysis of RNA sequencing of MDA-MB-231-MEK5-ca cells compared to parental controls demonstrating upregulation of EMT genes in MEK5-ca cells. qPCR for EMT markers in **(B)** MDA-MB-231 or (C) Hs-578T-vector and -MEK5-ca cells **(C)** Western blot of the epithelial marker CDH1 in MEK5-ca cells. **(D)** Transwell migration assay for MDA-MB-231- or Hs-578T-parental and -ERK5-ko cells. After 24 hours, migrated cells were fixed, stained with crystal violet, and quantified. Bars represent average number of migrated cells normalized to parental cells (set to 100%) ± SEM of triplicate experiments. * p < 0.05; ** p < 0.01; *** p < 0.001.

 We next sought to identify and characterize the pathways and processes by which MEK5 exerts pro-tumorigenic effects in TNBC. To address this, we employed two basal subtype TNBC cell lines (MDA-MB-231, Hs-578T). While these TNBC cells are categorized in the same molecular subtypes, they have distinct morphological and mutational profiles and both cell lines were included to account for cell type-specific differences. A constitutively active MEK5 expression construct (MEK5-ca) was generated in MDA-MB-231 and Hs-578T cells. Stable transfection of MEK5 overactivation was confirmed on a transcript (Figure 2A) and protein level (Figure 2B). Global transcriptome analysis of these MDA-MB-231-MEK5-ca cells was performed and identified genes that were differentially expressed (FC < 2, p<0.05) in the MEK5-ca expressing cells compared to the vector control. Gene Set Expression Analysis (GSEA) revealed a predicted increase in activation of the epithelial to mesenchymal transition (EMT) pathway in MEK5-ca cells when compared to control (Figure 3A). Consistent with these RNA-seq data, we observed an upregulation in expression of EMT associated factors FRA-1, SNAI1, MMP1, MMP2, and IL-8 in MDA-MB-231-caMEK5 cells compared to vector control (Figure 3B). Additionally, MEK5-ca increased expression of downstream MEK pathway members (MEF2A, MEF2C) and mesenchymal genes (CDH2, MMP2) (Figure 3C). MEK5-ca significantly downregulated the epithelial marker CDH1 gene expression (Figure 3C). Moreover, MEK5-ca cells showed greater migration potential, 3.25-fold (p < 0.01) of MDA-MB-231- and 1.24-fold (p < 0.05) of Hs-578T-vector cells (Figure 3D). These findings suggest that constitutive activation of MEK5 regulates the EMT/migration axis. 

**Figure 4:**
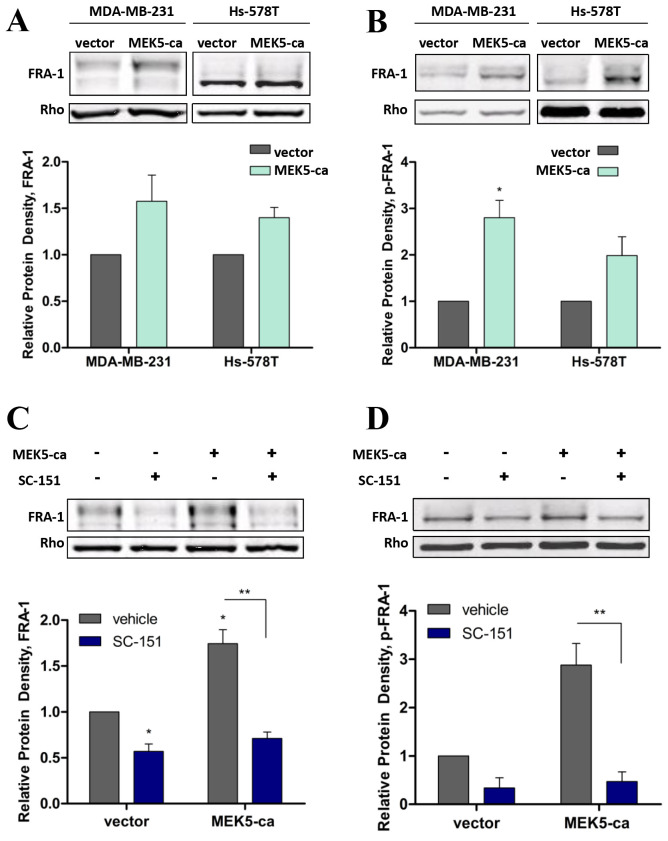
MEK5 regulation of FRA-1 expression. Western blot analysis of **(A)** FRA-1 and **(B)** p-FRA-1 expression in TNBC cells, n ≥ 2. Western blot analysis for **(C)** FRA-1 and **(D)** p-FRA-1 expression in Hs-578T-parental and -MEK5-ca cells treated with vehicle or SC-151 (1 μM) for 24 hours, n = 3. * p < 0.05; ** p < 0.01.

Our group, and others, have proposed FRA-1 to be a key downstream target of the MEK5/ERK5 pathway that regulates the EMT phenotype. We further characterized this relationship and showed that while constitutive activation of MEK5 does not increase total FRA-1, p-FRA-1 protein expression was significantly increased (Figure 4A, B). Furthermore, it was determined in both Hs-578T and MDA-MB-231 cells that MEK5-ca regulated the FRA-1 response to pan-MEK inhibition (MEK1/2 and MEK5 inhibition) with SC-151 (Figure 4C, D; Supplementary Figure 2). These data demonstrate that MEK5 functions through FRA-1 signaling activation.

###  MEK5 does not affect cell proliferation 

**Figure 5:**
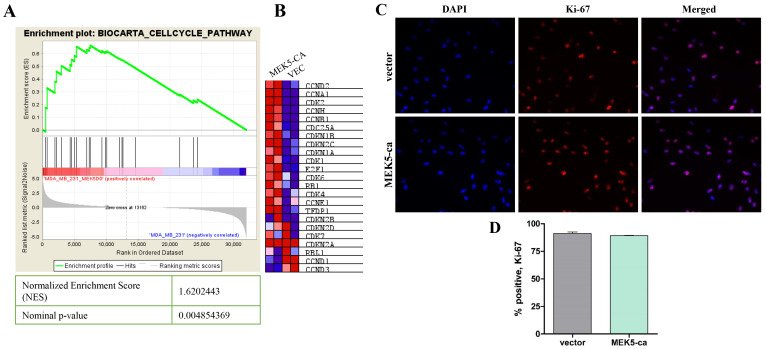
MEK5-ca upregulates cell cycle pathways but does not affect cell proliferation. **(A)** GSEA analysis of RNA sequencing of MDA-MB-231-MEK5-ca cells compared to parental controls demonstrating upregulation of **(B)** cell cycle related genes in MEK5-ca cells. **(C)** IF staining of Ki-67 in MDA-MB-231-vector and -MEK5-ca cells, viewed at 200x. **(D)** Representative images were taken per well and percentage of Ki-67-positive cells relative to total (DAPI-positive) cells was calculated. Bars represent mean % of Ki-67 positive cells ± SEM of triplicate experiments.

Gene expression analysis implicated MEK5 in cell growth and proliferation in both IPA disease and function analysis, as well as in GSEA canonical pathway analyses (Figure 5A, B). We next examined whether MEK5 activation promotes chemoresistance through increases in cell proliferation. We used Ki-67 staining to identify changes in proliferative capacity in the MEK5-ca cells compared with control in MDA-MB-231 cells. Contrary to expectation, we found that increases in MEK5 signaling alone did not induce increased proliferation in TNBC cells (Figure 5C, D). However, these cells are highly proliferative at baseline levels. We predicted that the activation of these cell cycle pathways might confer resistance to DNA damaging chemotherapeutics by way of maintaining active cell proliferation. We hypothesized that MEK5 does not confer increased proliferation but instead allows cells to sustain their proliferative phenotype even in presence of chemotherapeutic agents. This sustained proliferation may contribute to chemotherapeutic resistance in high MEK5 expressing TNBC tumors. 

## Discussion

 Given the diverse and integral regulatory functions of MAPK members and downstream targets in breast cancer progression and resistance, MAPK signaling pathways are promising additions to adjuvant chemotherapy regimens. Specifically, the MEK5/ERK5 pathway has emerged as a promising novel therapeutic target for breast cancer, as this signaling pathway regulates processes integral to breast cancer, including initiation, progression, metastasis, and drug resistance. Increased EMT and acquisition of mesenchymal features drives many of these processes, through maintenance of a cancer stem cell-like populations, promotion of cell motility and ultimately metastasis, and resistance to cytotoxic anticancer drugs. MEK5 signaling has been shown to promote EMT, cell survival, and evasion of apoptosis – mechanisms linked to adaptive resistance [28-32]. The efficacy of cytotoxic agents used in chemotherapy, the standard-of-care for various cancer types, is mitigated by activation of signaling pathways, such as MEK5, that confer drug resistance [29]. In basal-like breast cancer subtypes, overexpression of MEK5 in conjunction with ERK5 was associated with poor relapse- and metastasis-free survival in patients who received chemotherapy compared to patients not treated with chemotherapy, which suggests that MEK5-ERK5 expression could serve as a predictive marker for patient benefit from systemic treatments in the ER-negative breast cancer setting [24]. Moreover, in MDA-MB-231 cells ERK5 inhibition by TG02 augmented anti-cancer effects of chemotherapeutic agents conventionally used in TNBC treatment, including taxotere, vinorelbine, and cisplatin [22]. These results support the role of MEK5 signaling in regulation of survival and apoptosis and implicate MEK5 pathway involvement in chemoresistance [21]. Notably, our data suggested that MEK5 activation upregulated cell cycle pathways based on RNA sequencing analyses, but cell proliferation was not dramatically affected by MEK5/ERK5. These data are consistent with published data that have found MEK5-ERK5 signaling regulate cell cycle progression, notably by mediating G1/S transition through regulating cyclin D1 expression and through regulation of G2/M transition and is required for mitotic entry [33]. However, our data shows that MEK5 activation alone is not sufficient to increase proliferation in MDA-MB-231 cells. This supports the hypothesis that pathways affected by MEK5 activation may be cell type specific. This hypothesis is further supported by our findings that while constitutive activation of MEK5 increased migration of both TNBC cell lines, there were differences in the level of response amongst transfected Hs-578T and MDA-MB-231 cells. 

 Interestingly, when MEK5-ca cells were treated with the MEK1/2/5 inhibitor SC-1-151. Although previously observed cellular activity of SC-151 is consistent with computationally anticipated type III MEK5 inhibitor affinity [34, 35], observations in this report examining activity of SC-1-151 against constitutively active mutant MEK5DD suggest that there may be additional interactions beyond type III MEK5 interactions. Classically, type III inhibitors displace the c-helix and prevent MEK activation via dual phosphorylation on the TEY motif. There has, however, been an increasing awareness [36-41] that type-III kinase binders may induce additional allosteric modification beyond displacement of the C-helix. There also exists the possibility that SC-1-151 may have interactions at other MEK5 allosteric sites, may modify a MEK5 protein/protein interaction, or have activity at a yet uncharacterized protein. Further experiments are necessary to establish interactions at the molecular level through clinical relevance to fully evaluate these observations. These findings demonstrate the need for a greater understanding of MEK5 inhibition; assays contributing to this understanding are actively in development in our collaborative laboratory groups. 

 While a link between activated MEK5/ERK5 signaling and EMT has been demonstrated [8, 9, 28, 42], specific downstream substrates that are responsible for this activity remains understudied. EMT transcription factors (EMT-TF), including the SNAIL, TWIST, and ZEB families, as well as c-Fos and Fra-1 promote the acquisition of a mesenchymal phenotype. EMT-TFs regulate cell plasticity and are involved in many stages of cancer progression such as initiation, primary growth, invasion, dissemination, metastasis, and drug resistance [43-45]. Within subsets of metastatic breast cancer, EMT-TFs are associated with poor prognoses and increased risk of metastatic outcomes [44, 45]. In this study, we found the MEK5/ERK5 signaling activates the EMT-TF Fra-1, which is responsible for driving a mesenchymal and migratory cell phenotype. These findings provide supportive evidence consistent with prior studies that activation of MEK5 upregulates breast cancer cell proliferation, migration and promotes the mesenchymal phenotype through FRA-1 signaling activation. 

## Materials and Methods

### Reagents and cell culture 

 MDA-MB-231 and Hs-578T cell lines, both categorized as mesenchymal-like TNBC cells of the basal intrinsic subtype with different molecular characteristics [46], were acquired from American Type Culture Collection. Both cell lines were cultured in Dulbecco’s Modified Eagle Medium (DMEM; pH 7.4; Invitrogen, Carlsbad, CA) supplemented with 10% Fetal Bovine Serum (FBS; Hyclone, Salt Lake City, UT), 1% non-essential amino acids, minimal essential amino acids, sodium pyruvate, antibiotic/anti-mycotic and insulin under mycoplasma-free conditions at 37°C in humidified 5% CO2 and 95% air. Dimethylsulfoxide (DMSO) was purchased from Fisher Scientific (Waltham, MA). Dosing for SC-1-151, which inhibits MEK1, MEK2 and MEK5, kindly provided by Patrick Flaherty (Duquesne University, Pittsburgh, PA), was 1 μM for *in vitro* studies unless otherwise indicated. While cells were maintained in 10% FBS-containing DMEM as described above, for drug treatment experiments cells were placed in low serum media, or phenol-free media supplemented with 5% charcoal-stripped FBS, Glutamax (ThermoFisher Scientific, #35050079), non-essential amino acids, minimal essential amino acids, and penicillin-streptomycin. 

### Generation of constitutively active MEK5 TNBC cells 

The constitutively active pcDNA3-MEK5(DD) (MEK5-ca) expression plasmid, graciously donated by Marcus Buschbeck (Max-Planck-Institute of Biochemistry, Martinsried, Germany), was produced by site-directed mutagenesis replacing S311 and T315 by aspartate (D). TNBC cells were plated in 10 cm dishes and allowed to adhere overnight at 37°C. Cells were transfected with 5 μg of plasmid in 300 μL Opti-MEM. Transfection was accomplished using 15 μL Attractene per manufacturer’s instructions (Qiagen, Valencia, CA). Media was changed the following day and cells were treated with selectable marker every two days. Once stable cells were obtained, viable colonies were cloned (MDA-MB-231) or pooled (Hs-578T). Stable expression was confirmed by qPCR and Western blot. 

### Quantitative polymerase chain reaction (PCR) 

Cells were grown in phenol red-free DMEM supplemented with 5% charcoal-stripped (CS) fetal bovine serum (5% CS-DMEM) for 48 hours and treated with compounds. After 24 hours, cells were collected and total RNA was extracted using the Quick RNA Mini Prep Kit in accordance with the manufacturer’s protocol (Zymo Research, Irvine, CA). The quality and concentration of RNA were determined spectrophotometrically by absorbance at 260 and 280 nm using the NanoDrop ND-1000. Total RNA (1 μg) was reverse-transcribed using the iScript kit (BioRad, Hercules, CA) and qPCR was performed using SYBR-green (Bio-Rad Laboratories, Hercules, CA). Cycle number was normalized to β-actin and vehicle-treated cells scaled to 1, n = 3. For patient-derived xenografts, RNA was isolated from tumor pieces using QIAzol Lysis Reagent (Qiagen, Valencia, CA) and Quick RNA Mini Prep Kit (Zymo Research, Irvine, CA). 

### Western blot

Cells were cultured in 10% FBS-supplemented DMEM. At confluence or post 24-hour treatment, cells were collected in PBS, pelleted, and lysed with mammalian protein extraction reagent (MPER) supplemented with 1% protease inhibitor and 1% phosphatase inhibitors (I/II) (Invitrogen, Grand Isles, NY). Samples were centrifuged at 12,000 RPM for ten minutes at 4°C to obtain supernatant containing protein extracts. NanoDrop ND-1000 was used to determine protein concentration of samples by absorbance at 260 and 280 nm. After proteins were heat-denatured at 100°C on a heating block, 40 μg of protein was loaded per lane on Bis-Tris-nuPAGE gel (Invitrogen, Grand Isles NY). Protein was then transferred to nitrocellulose membranes using iBlot and iBlot transfer stacks per manufacturer’s instructions (Invitrogen, Grand Isles, NY). Membrane was incubated at room temperature with 5% bovine serum albumin (BSA) in 1% Tris-buffered saline, 0.1% Tween 20 (TBS-T) for 1 hour to block non-specific binding followed by 4°C incubation overnight with primary antibodies (MEK5: anti-rabbit, Santa Cruz, 10795; p-FRA-1 (Ser 265), Cell Signaling Technology, 3880; FRA-1 (D80B4), Cell Signaling Technology, 5281). After three 15-minute washes in 1% TBS-T, membranes were incubated with appropriate secondary antibodies for at least one hour. IR-tagged secondary antibodies were purchased from LiCor Biosciences (Lincoln, NE) and used at a 1:10,000 dilution in 5% BSA. Following incubation with secondary antibodies, membranes were washed three times for 15 minutes per wash in 1% TBS-T, and blots were analyzed by the Odyssey Infrared Imaging System (LiCor Biosciences). Band density was quantified by LiCor gel imager. Data were normalized to Rho GDI-α (Santa Cruz Biotechnology, Santa Cruz, CA), serving as loading control. Experiments were conducted in triplicate with representative blots shown.

### Transwell migration assay

Cells (2.5 x 10^4^) in 500 μL phenol red-free Opti-MEM were seeded in the upper chamber of a 24-well transwell chamber. 5% DMEM was used as a chemoattractant in the lower wells. Phenol red-free Opti-MEM was used in one well as a negative control to assess basal migration rates. After 24 hours, inner membranes were scrubbed to remove non-migrated cells. Cells on the outer membranes were fixed in formalin and stained with crystal violet. Membranes were excised from the transwell insert and mounted on glass slides. Number of migrated cells were visualized by microscopy and counted. Bars represent percent control migrated cells per 200x field of view ± standard error of mean (SEM) for triplicate experiments. 

### Immunofluorescent staining

Cells were seeded in 96-well plates at a density of 2,000 cells per well. For morphometric analysis, cells were fixed in formalin 3 days after drug treatment and permeabilized with Triton X-100 (Sigma, St. Louis, MO, USA). The cytoskeleton was identified with Alexa Fluor® 555 to visualize phalloidin (1:200; Cell Signaling Technologies). Cells were counterstained with DAPI (1:1000; Invitrogen). ApoTome fluorescent images were taken on an inverted microscope (Zeiss, Thornwood, NY) and digitally filtered to obtain optical slices. For Ki-67 analysis, cells were fixed in-well and stained with Alexa Fluor® 555 conjugated to Ki-67 (1:200; BD Biosciences). 5 images per well were captured at 400x, n = 3. Results are represented as percent positive Ki-67 staining (red) of total number of cells visualized by DAPI (blue). 

### Whole genome sequencing and pathway analysis 

MDA-MB-231 transfected cells were extracted for total RNA. Changes in gene expression were determined using next generation sequencing as described [46]. Genes significantly up-regulated in both cell lines were pooled and uploaded into the online pathway interaction database (PID) [ http://www.cancer.gov], followed by analysis of significantly down-regulated genes. Based on -log(p-value) calculated from output data, top regulated pathways were determined. 

### Statistical analysis 

Statistical analyses were performed using Graphpad Prism software (Graph-Pad Software, Inc., San Diego, CA). Data were subjected to unpaired Student’s t-test, with p-value < 0.05 considered statistically significant. Studies involving more than two groups were analyzed by one-way analysis of variance (ANOVA) followed by Tukey’s post-hoc multiple comparison tests. *, p < 0.05; **, p < 0.01; ***, p < 0.001. 
